# Competition, Conflict and Change of Mind: A Role of GABAergic Inhibition in the Primary Motor Cortex

**DOI:** 10.3389/fnhum.2021.736732

**Published:** 2022-01-04

**Authors:** Bastien Ribot, Aymar de Rugy, Nicolas Langbour, Anne Duron, Michel Goillandeau, Thomas Michelet

**Affiliations:** ^1^CNRS, IMN, UMR 5293, University of Bordeaux, Bordeaux, France; ^2^CNRS, EPHE, INCIA, UMR 5287, University of Bordeaux, Bordeaux, France; ^3^Unité de Recherche Clinique Intersectorielle en Psychiatrie à Vocation Régionale du Centre Hospitalier Henri Laborit, Poitiers, France; ^4^Faculté de Médecine, Université de Paris, Paris, France

**Keywords:** decision, executive control, behavioral adaptation, reaching movement, transcranial magnetic stimulation, silent period, inhibition, primary motor cortex (M1)

## Abstract

Deciding between different voluntary movements implies a continuous control of the competition between potential actions. Many theories postulate a leading role of prefrontal cortices in this executive function, but strong evidence exists that a motor region like the primary motor cortex (M1) is also involved, possibly *via* inhibitory mechanisms. This was already shown during the pre-movement decision period, but not after movement onset. For this pilot experiment we designed a new task compatible with the dynamics of post-onset control to study the silent period (SP) duration, a pause in electromyographic activity after single-pulse transcranial magnetic stimulation that reflects inhibitory mechanisms. A careful analysis of the SP during the ongoing movement indicates a gradual increase in inhibitory mechanisms with the level of competition, consistent with an increase in mutual inhibition between alternative movement options. However, we also observed a decreased SP duration for high-competition trials associated with change-of-mind inflections in their trajectories. Our results suggest a new post-onset adaptive process that consists in a transient reduction of GABAergic inhibition within M1 for highly conflicting situations. We propose that this reduced inhibition softens the competition between concurrent motor options, thereby favoring response vacillation, an adaptive strategy that proved successful at improving behavioral performance.

## Introduction

Because most decisions are implemented through concrete action, understanding their neuronal bases requires to address eventually the question of competition between potential responses. Among the several tasks designed for studying response competition, those designed to produce a conflict gave rise to a strong interest because they offer distinct cognitive conditions with sharply defined chronometric and psychometric behavioral evidences (Smith, 1968; MacLeod, 1991).

Among the numerous and distributed brain areas involved in motor decisions (Cisek, 2012), it is now well admitted that primary motor cortex (M1) does not only operate as a pure output in the motor process, but also contributes to the integration of cognitive variables that influence and bias movement execution (Georgopoulos, 2000). It is thus expected that this core brain region plays an active role in the conflict decision process (Michelet et al., 2010; Klein et al., 2014), in addition to other frontal and prefrontal regions like the anterior cingulate cortex emphasized by the classic conflict-monitoring theory(Botvinick et al., 2004).

Among several studies that helped to better understand the role of M1, those using transcranial magnetic stimulation (TMS) are of particular interest because they associate both excellent spatial and temporal resolution with a direct access on the motor output function. The majority of these studies (Leocani et al., 2000; Michelet et al., 2010; Klein et al., 2012) used single pulse TMS during the RT period, defined since Donders as the time elapsing between the onset of the stimulus and the onset of the response, and consequently considered to represent the time during which a decision is made (Meyer et al., 1988). Thanks to precise analyses of the amplitude of motor evoked potentials (MEP) that reveal the corticospinal excitability (CSE), they generated a great deal of knowledge about the chronometry and the network organization of the cortical mechanisms involved in conflict. Importantly these studies strongly suggest that M1 is not just a blind executor of a decision made upstream but is also biased and modulated by cognitive influences during the RT period (Michelet et al., 2010; Klein et al., 2014).

However, at least in complex behaviors, decision is not always limited to the RT period and the decision process is supposed to evolve after movement onset, as suggested by behavioral adjustment or correction during ongoing execution of movement (Rabbitt and Vyas, 1981).

Hence it seems of particular interest to further study the involvement of M1 during movement execution, and more particularly to address the inhibitory processes that are thought to shape movement execution (Bari and Robbins, 2013). Among the different possibilities allowing to measure inhibition within the central nervous system, the silent period (SP) is of particular interest as it must be elicited during voluntary electromyographic (EMG) activity (Ziemann et al., 1993; Chen et al., 1999). More particularly, this SP is characterized by a pause in the EMG signal whose cortical origin has been clearly proven (Chen et al., 1999). Although debate still exists on a spinal contribution to its earliest component (Schnitzler and Benecke, 1994), the SP is proven to be a reliable measure of GABA intracortical inhibition (Siebner et al., 1998). Unfortunately, the vast majority of studies interested in conflict have used experiments involving ballistic-like movements (e.g., quick button presses mediated by adduction/abduction of the index finger), lacking the biomechanical complexity of most natural actions as well as post-onset control requirements.

To fill this gap, here we designed a novel conflict task in order to assess the potential role of cortical inhibition during ongoing movement execution. We based our paradigm on a center-out reaching task which, contrary to simple categorical decision, is subject to factors such as effort, biomechanical complexity, and ongoing movement control (Cos et al., 2011; Cos et al., 2012; Coallier et al., 2015).

**FIGURE 1:**
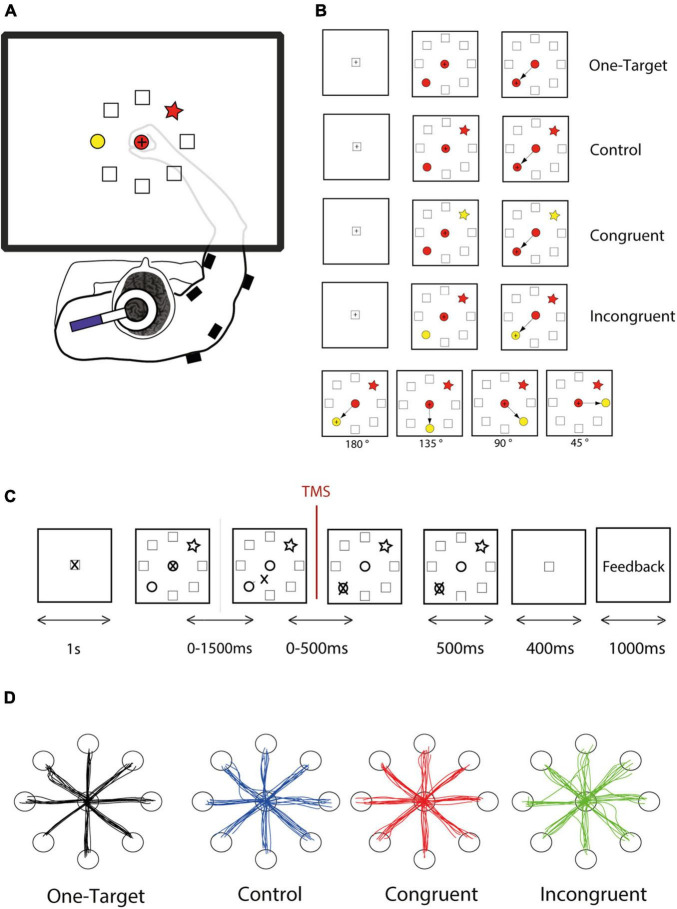
Experimental design. **(A)** The subject responded to the task by moving a cursor on a digitizing tablet. Stimuli and cursor feedback are projected onto a mirror placed between the table and the monitor. Single-pulse transcranial magnetic stimulation (TMS) was applied over left primary motor cortex, and EMG activity was measured thanks to wireless electromyographic surface electrodes attached to the main muscle groups of the right upper arm. **(B)** The task follows a shape-matching rule, and color dimension is an irrelevant feature of the stimuli. However, shapes and colors were assembled to form different cognitive situations, of three different levels of difficulty. For each cognitive condition, the target could be located in every one of 8 positions arrayed in a circle, and the distractor could be spaced from 45 to 180°. **(C)** The DSLT task began (after a 1,000-ms rest period during which the cursor rests in the home position) with the simultaneous presentation of one central shape and the peripheral target and distractor. The subject had up to 1,500 ms to initiate the movement and up to 500 ms to reach the correct target and stay inside for 500 ms. Then a positive or negative visual feedback, depending on the response accuracy appeared for 1,000 ms. **(D)** Examples reach trajectories in one-target, Control, Congruent, and Incongruent conditions from one subject.

For this pilot study, twenty subjects performed our directional Stroop-like task (DSLT) allowing to study a broad range of competition level and conflict situations. Two potential targets arrayed randomly in a circle are presented simultaneously with a central cue. The choice is imposed by a simple shape-matching rule, but the color feature of the cues is manipulated to provide an irrelevant and conflicting dimension, thereby allowing to generate three cognitive situations, corresponding to control, congruent, and incongruent conditions (Figures 1A,B). Additionally, we also carefully controlled the angular distance between the target and distractor in order to influence the competition effect within each condition (Figure 1B).

This DSLT coupled with TMS allowed us to address specifically the role of gabaergic inhibition during post-onset decision period within the primary motor cortex. We hypothesized that, in addition to pre-movement (RT), conflict can still influence the decision process within the M1 during the ongoing movement.

As a whole, the present experiment aimed at addressing the following questions:

(1) Is the DSLT a valid task to study behavioral impact of response competition and conflict?
(2) Is conflict resolution based on the same neuronal processes as other competition for the selection of a target between several options?
(3) Can the inhibitory activity within the primary motor cortex account for the behavioral results found in a complex conflict task?

This study will incidentally provide information on the general concept of response competition and on the peculiar role of incongruent situations, appealing for a necessary clarification of the terms “competition” and “conflict” to prevent research’s misdirection.

## Materials and Methods

### Participants

Twenty subjects (9 females), mean age 24.2 (±3.4) participated in the experiment. All had normal or corrected-to normal visual acuity, were right-handed according to the Edinburgh Handedness Inventory (Oldfield, 1971), and were free from any contraindication to transcranial-magnetic stimulation (TMS; Rossi et al., 2009). The experimental procedure was approved by the national ethics committee (CPP N° 2013A01444936), and was carried out in accordance with the principles of the revised Helsinki Declaration [World Medical Association General Assembly (WMAGA), 2008]. All subjects gave written informed consent prior to the experiment and were financially compensated for their participation.

### Apparatus

The task apparatus consists of a digitizing tablet (GTCO Calcomp, Columbia, MD, United States; 0.915 × 0.608 m) and a half-silvered mirror suspended 16 cm above and parallel to the digitizer plane. Visual stimuli were projected onto the half-silvered mirror by an LCD monitor suspended 16 cm above the mirror, producing the illusion that the targets lie on the plane of the digitizing tablet. Subjects made reaching movements in the horizontal plane using a digitizing stylus (moving the cursor) embedded within a 3D-printed cylinder held by the subject’s right hand. The semi-silvered mirror was such that the hand was mostly invisible to subjects, who mostly saw the cursor they controlled and the visual stimuli delivered to them (Figure 1A). The stylus position was sampled at 125 Hz with a spatial resolution of 0.006 ± 0.127 mm. The control of the task, stimulus display, and synchronization of task events and signal recording were performed by a custom written LabVIEW program (National Instruments, Austin, TX, United States). The data were stored in a Matlab structure and analyzed using custom Matlab scripts (Mathworks, Natick, MA, United States).

### Task Design

We designed a new experimental paradigm based on a center-out reaching task combined with an analog of the original Stroop test. Because of the combination of these two features, we called this task the DSLT (for Directional Stroop like task). The principle used here is the shape-matching protocol in which the subject had to select the peripheral target with the same shape as the central cue, whatever the colors filling these shapes (Figure 1). The color of the stimuli is consequently an irrelevant information. Using different combinations of color and shape, we were able to assess interference between 2 competing tendencies (shape or color association), and consequently present three cognitive conditions comparable to those of the classical Stroop: control, congruent, and incongruent conditions (Figure 1B). In the control condition, all the shapes are filled with the same color (e.g., red, Figure 1B), such that the color is not supposed to bias the movement toward one particular peripheral shape. In the congruent condition, the target is colored with the same color as the central cue (e.g., red) while the distractor is filled with another color (e.g., yellow), such that the color should bias the movement toward the target. Finally, in the incongruent condition, the distractor as the same color as the central cue (e.g., red), such that the color should bias the movement toward the distractor. Because the cue is in the central position and the target in 1 of 8 potential positions at the circumference of a 12 cm radius circle (equidistantly arranged, at 45° intervals), this task involved a wide range of movement directions. Moreover, the relative distance between the target and the distractor can also be easily manipulated in this task (Figure 1B, bottom panel).

Task stimuli were manipulated and presented in a pseudo random order in order to satisfy the following constraint: (1) control, congruent, and incongruent trials were presented in equivalent proportion; (2) similar number of trials for the eight movement directions (3) similar number of trials for angular distance between target and distractor (i.e., 45, 90, 135, and 180°) (4) similar number of trials using the red or yellow color to fill the central cue, and (5) similar number of occurrence for each of the four shapes (triangle, star, square, or heart symbol). Each shape has an equivalent surface and are filled either with a red or yellow color with equal luminance.

**FIGURE 2:**
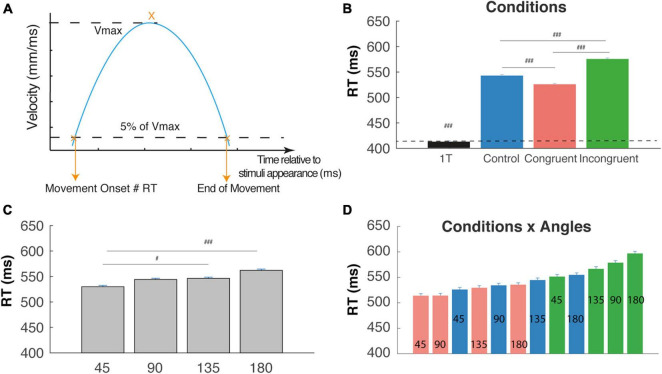
Reaction time (RT). **(A)** RT corresponds to the movement onset time relatively to the appearance of the cues; it is defined by the time at which the velocity cross a threshold defines as 5% of the maximal tangential velocity (Vmax). **(B)** RT are significantly shorter for the one-target condition in comparison with two targets cognitive conditions. RT trials are significantly longer than control and congruent trials RTs (interference effect) and Congruent trials are faster than control trials (congruency effect) (###*P* < 0.0001). **(C)** RT increase with the angular distance between targets. **(D)** Almost linear increase of RT when considered the (12) conditions x angles trial types and revealing a continuum in the competition level induced by these trials-types. ^#^*p* < 0.01.

Each trial comprised the same succession of events (Figure 2C). A trial started when the cursor is within the home position represented by a square at the center, followed by a 1 s rest period during which the subject was instructed to remain still. Next the task itself (DSLT) appeared, i.e., the simultaneous presentation of one central shape and the peripheral target and distractor, followed by the response period during which the hand leaves the home position to reach the peripheral target. The evaluation period ended the trial with the appearance of a positive or negative visual feedback. Subjects had 1,500 ms to leave the home position, and 500 additional ms to reach the target. Despite this temporal constraint which was easy to comply with, subjects were instructed to move as quickly and accurately as possible. The trial aborted if the hand position cursor was moved outside an acceptable diameter of the central cue (±10% of the cue diameter) before the target and distractor appearance.

In addition to the DSLT, a one-target task was also performed to obtain control values for behavioral variables as well as SP duration when no choice had to me made (see below: RT, initial deviation, and SP). All reaching curvature and dispersion measures were normalized for each direction by those control values obtained in the one-target experiment. Each subject performed one session that comprised 657 trials (with a short pause every 100 trials) and lasted about 2 h, including the threshold hunting phase. After a first series of 200 trials without TMS, TMS was applied for each trial. The three trial conditions and the four angles were interleaved in a pseudorandom order, and the mean number of SP in a single muscle computed for each of these 12 different experimental conditions was 31 per subject. Eighty trials were also used for measuring the SP in the One Target condition in the eight different direction, with 10 SP for each movement direction and for a single muscle.

### Electromyographic, Motor Evoked Potential, and Silent Period Recording

The silent period (SP) is induced when a TMS pulse is applied during an ongoing movement execution, and corresponds to a transient suppression of the EMG activity. The TMS pulse was applied 30 ms after the cursor left the central zone (2 cm diameter) in order to stimulate during an active EMG period, yet soon after movement execution (but in every trial, after the 100 ms following action onset, hence allowing to measure the initial deviation in the absence of TMS perturbation).

Surface electromyographic recording was performed in nine muscles, eight of each chosen on the basis of their involvement in the reaching movements (Supplementary Figure 1): biceps long head, biceps short head and anterior deltoid as flexors, triceps brachii and posterior deltoid as extensors, pectoralis major as arm adductor, lateral deltoid as arm abductor and trapezius as shoulder elevator. Finally, the FDI (a finger abductor) was recorded in order to measure the resting motor threshold (RMT). EMG activity was acquired with a Trigno™ Wireless EMG Systems (Delsys Inc., Boston, MA, United States) amplified (by a factor of 909), band-pass filtered (Bandwidth 20 ± 5 Hz, >40 dB/dec), digitized on line (rate 2 kHz), and later rectified and integrated.

During experimental recording sessions, subjects were seated in a chair. Their heads were slightly immobilized on the right to counteract the pressure exerted by the TMS coil positioned over the left M1, and their left arm rested on their knees. For the first twelve subjects, a figure- of-eight coil (Double 70 mm Coil, Magstim Company Ltd., Whitland, Dyfed, United Kingdom) was used to stimulate M1 over the left hemisphere. During this first part of the experiment, the duration of the SP obtained were too short to be confidently attributed to a cortical inhibition (mean duration: around 60 ms, but see the section “Discussion” and Supplementary Material). In order to increase the SP duration, and because the effect of simulation intensity on the SP duration is well documented (Taylor et al., 1997; Säisänen et al., 2008), we used for the remaining eight subjects a circular coil (90 mm Coil, Magstim Company Ltd., Whitland, Dyfed, United Kingdom) with a larger surface of stimulation allowing to stimulate a larger region, with a higher intensity, corresponding to the forelimb region of the primary motor cortex. The rest of the protocol was exactly the same regardless of the coil used. In the following section, the results correspond to the circular coil experiment, while the results obtained with the figure- of-eight coil are presented in Supplementary Material. The coil was held tangentially on the left hemi-scalp with its handle pointing backward at an angle of about 45 degrees from the midsagittal axis. The resting motor thresholds were established using the criterion of lowest intensity of stimulation that allowed to induce peak-to-peak amplitude MEPs at rest of approximately 100 μV (in at least 8 of 10 consecutive trials) in the FDI muscle of the right hand (Supplementary Figure 2). In order to avoid any modification of MEPs amplitude due to background EMG activity, trials in which muscular pre-activation was greater than 100 μV within a 500 ms window preceding the TMS pulse were discarded. Then, the optimal scalp position (OSP) of the coil was defined as the position allowing to induce MEPs simultaneously in the 8 muscles involved in the reaching movements, which is possible because of the proximity of the representation area of the studied muscles. The OSP was obtained by moving the coil in approximately 0.5 cm steps around the subject’s left M1. Throughout the experiment, the coil was manually maintained over the OSP using the Brainsight frameless stereotactic system (Rogue Research Inc., Montreal, Canada) to continuously monitor coil placement; coordinates of each stimulation relative to the hotspot were recorded for *post hoc* verification. The stimulus intensity was set at 120% of the resting motor threshold in order to obtain SP in the recorded muscles (see Supplementary Figure 2).

### Analysis

Data analysis was carried out offline using custom written Matlab programs.

**FIGURE 3:**
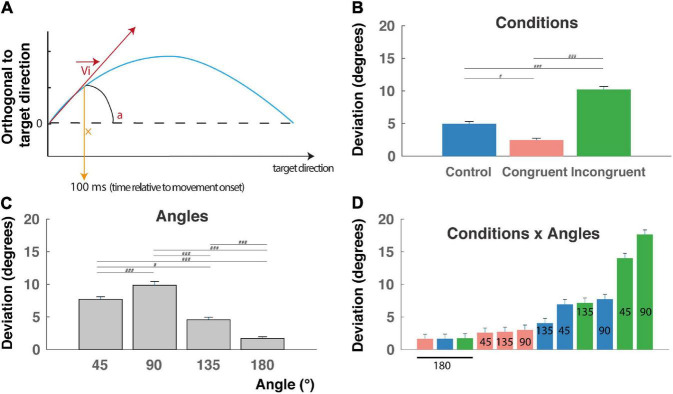
Initial deviation (ID). **(A)** The initial deviation (ID) corresponds to the angular difference between the initial direction computed 100 ms after the movement onset and the overall direction determined as the center of the target location. It indicates the decision made early after movement onset. Blue line: reach trajectory. Red arrow: initial direction. **(B)** ID for incongruent trials are significantly longer than control and congruent trials RTs and congruent trials are faster than control trials (^###^*P* < 0.0001) indicating that the interference and congruency effects are found also after movement onset. **(C)** ID decrease with the angular distance between targets, except for the 90° angular separation. **(D)** Almost linear increase of RT when considered the (12) conditions x angles trial types and revealing a continuum in the competition level induced by these trials-types also after movement onset (but see text for peculiarity of the 90° and 180° angular distances). ^#^*p* < 0.01.

Movement onset was defined by the time at which the cursor tangential velocity exceeded 5% of the maximal tangential velocity (Figure 2A), and RT corresponded to the time between cue appearance and movement onset. Calculation of the initial deviation was based on Ludwig and Gilchrist (2002): a rotation was applied on the reach trajectory so that the straight line between start and endpoint always coincided with the horizontal axis, and the values on the ordinate indicate the perpendicular deviations from the straight line (Figure 3A). We then calculated the initial deviation (ID) as the angle between the horizontal axis and a fixed point in the movement trajectory obtained 100 ms after the movement onset.

Most of these movements were single-curved trajectories, whereby lateral deviations from target direction display a single peak. However, for some trials, the hand trajectory changed directions multiple times during the movement, often characterized by a double-peak curve of lateral deviations indicating movement corrections. A careful examination was conducted on each trial and when more than one local maxima (peak) was found on the trajectory, we classified the trial as “self-corrected” or vacillation (see Supplementary Figure 4C for examples).

**FIGURE 4:**
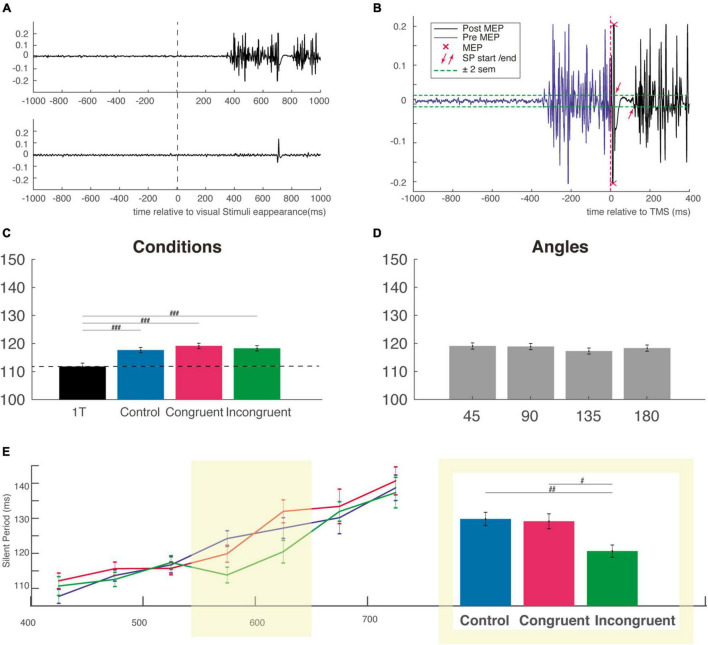
Silent period duration (SP). **(A)** Typical EMG response in a muscle involved (top EMG trace) or not (bottom EMG trace) in a reaching trial. Activity is centered on the onset of visual stimuli **(B)** example of calculation of the SP for the EMG trace shown in panel **(A)** the SP was measured from offset of the MEP to the resumption of voluntary EMG activity. **(C)** The SP is not modulated by the cognitive condition but is shorter for the one-target condition, attesting the influence of the very competition between potential options. **(D)** SP duration as a function of the angular distance between the target and distractor. **(E)** Mean SP in each bin of RT as a function of cognitive condition (control, congruent, and incongruent) showing that SP generally increase with competition strength. The two bins centered, on the mean incongruent RT, is highlighted in yellow and the SP for this subgroup is presented in the adjacent inset (the same ordinate axis as for the main graph). MEP, motor evoked potential; sem, standard error of the mean. ^#^*p* < 0.01; ^##^*p* < 0.001; ^###^*p* < 0.0001.

For the measurement of the silent period duration, we considered the absolute silent period. Its beginning is defined as the first moment after MEP the EMG crossed the rest period ± 2 sem. It ends, similarly, at the first moment after the suppression of the ongoing EMG, when the EMG crossed the rest period ± 2 sem (see Figure 4B). A first automatic analysis is performed with a custom Matlab program and verified visually for each trial. The duration of the silent periods is not influenced by changes in contraction strength (Taylor et al., 1997), rendering unnecessary to normalize the SP to background EMG.

Because the use of a supra-motor threshold could have disturbed motor execution, movement times were not presented in detail in this paper, and we focused our behavioral analyses on RT and Initial deviation that both occurred before the TMS pulse.

Because the silent period is defined as a suppression of ongoing EMG activity, it can only be recorded when muscles are actually activated for a particular reaching movement. This activation was characterized when the EMG activity before at least 100 ms preceding the TMS pulse exceeded by ± 2SD the baseline EMG level recorded during the rest period (green dotted line; Figure 4B). As all muscles do not contribute to each reaching movement, this means that all muscles did not exhibit a SP for every reaching direction. Hence, we considered a muscle only if the SP were consistently found in at least 8 out of 10 times for this movement in the one-target condition. The SP duration for a movement direction corresponds to the mean of the SP actually recorded in each considered muscles.

ANOVAs and *t*-tests with Bonferroni-Dunn correction for *post hoc* analysis were used. We set the significance levels for the ANOVAs to correct for multiple comparisons and for the *post hoc t*-tests to *P* < 0.05. All data are given as means ± SE. For RTs and SPs, we also performed a one-way ANOVA, reporting the statistical size effect index *f* [see (Cohen, 1988) for more information] including the One-Target condition. Indeed, in the One-target condition the target location is identified unambiguously by its spatial location, while in the three cognitive conditions two potential targets (target and distractor) are simultaneously presented. Hence, the comparison between the One-target condition (no competition) and all other cognitive conditions (competition between representations of the alternative choices) is a good indicator of the very impact of competition on RTs or SPs. This One-target condition was excluded from the two-way ANOVA with Angle separation as a factor because no distractor (and consequently no angle) was used in this condition. Finally, the One-Target condition is not directly presented in the ID analyses because each trajectory in a particular direction was normalized relatively to the correspondent mean trajectory of the one-target trials.

## Results

### Behavioral Results

Because of the extremely low error rate (<3%), only behavioral measures for correct trials were considered in the analyses. Regarding the behavioral analyses, we only analyzed in the following section the behavioral parameters sampled before the stimulation time, and consequently not affected by the TMS pulse. We are then able to pool RTs or initial deviations (IDs) analyses for trials *with* and *without* TMS. For the statistical analysis of RTs, IDs and silent periods (SPs), two factors were taken into account: *task conditions* (control, congruent, or incongruent) and *angular distance* between the target and distractor (45°, 90°, 135°, and 180°).

We first performed a one-way ANOVA on RTs in order to compare with the One-Target condition. This analysis showed a clear main effect of condition on RT (*F*_(3,12754)_ = 1268.6, *P* < 0.0001), *f* = 0.18 as confirmed by *post hoc* comparison showing that mean RT was significantly lower for the One-target condition (412 ± 1.7 ms) than the other three cognitive conditions, confirming a strong effect of competition on RTs. Moreover, we found, respectively increased RTs for incongruent trials (574 ± 2 ms) and decreased RTs for control trials (541 ± 1.8 ms) as compared to congruent (524 ± 1.7 ms) trials, attesting the congruency and interference effect (Figure 2B, *t*-test, *P* < 0.00001). We also found a clear main effect of angular distance on RT (*F*_(3,9009)_ = 32.97, *P* < 0.0001), *f* = 0.1, as confirmed by *post hoc* comparison showing that mean RT was significantly lower for 45°, and increased until 180° (Figure 2C, 530 ± 2.4; 544.1 ± 2.3; 546.3 ± 2.3; 562 ± 2.5; *t*-test *p* < 0.01). Finally, a significant *Condition* X *Angle* interaction (Figrue 2D) was found (*F*_(6,9009)_ = 3.71, *P* < 0.002), *f* = 0.05, indicating a continuum of increasing RT from the congruent-45° trials to the incongruent-180° trials that could be due to gradual increases in the competition process between targets and distractors before movement onset. *Post hoc* tests are not described here in detail, because of the numerous significant differences between trial types (56 out of 66 tested comparisons).

This behavioral congruency effect and interference effect found in the RT are thought to reflect the competition between the two incompatible responses induced either by the color or by the shape of the cue. This hypothesis was further confirmed by a main effect of condition found on IDs (Figure 3A; *F*_(2,9009)_ = 117.14, *P* < 0.0001), *f* = 0.16, with *post hoc* comparisons indicating higher deviation toward the distractor for the incongruent condition (10.2 ± 0.4 mm), intermediate deviations for the control condition (4.9 ± 0.3 mm), and lower deviations for the congruent condition (2.5 ± 0.2 mm) (Figure 3B, *P* < 0.00001 for all *t*-tests). We also found a clear main effect of angular distance on IDs (*F*_(3,9009)_ = 71.93, *P* < 0.0001), *f* = 0.15, confirmed by *post hoc* comparisons showing that mean ID was significantly higher for 45° (7.7 ± 0.4) and decreases for 135° (4.6 ± 0.4 mm) and 180° (1.7 ± 0.2 mm) (Figure 3B, *P* < 0.01 for all *t*-tests), with a higher value for 90° (9.8 ± 0.6 mm), probably due to geometrical factors (see the section “Discussion”). Finally, a significant *Condition* X *Angle* interaction was found (*F*_(6,9009)_ = 22.78, *P* < 0.0001), *f* = 0.12, indicating a continuum of increasing ID from the congruent-45° trials to the incongruent-180° trials, and thereby could be a continuum in the competition process occurring after movement onset. Unsurprisingly, ID for a target localized at 180° of the distractor exhibited the lower IDs for geometrical reason (see the section “Discussion”). *Post hoc* interactions are not described here in detail, because of the numerous significant differences between trial types (51 out of 66 tested interactions).

The percentage of trials during which the hand trajectory changed course during the movement was calculated. In a significantly greater proportion of incongruent trials (19.3%), the hand trajectory changed course during the movement, indicating a self-correction, in comparison to control (11.6%) and congruent (6.7%) trials (X2 = 14.66, *p* < 0.0001; Supplementary Figure 4).

### Silent Period Duration

The mean intensity of a single TMS pulse needed to evoke a MEP of 100 μV at rest in the FDI, considered as the resting motor threshold, was 51 ± 3% of the stimulator output for the experiment with the figure-of-eight coil and 47 ± 3% with the circular coil.

We next considered how the SP duration was influenced by the competition, the congruency and interference effect as well as by the angular distance between target and distractor. Using a one-way ANOVA on SP obtained in our different conditions (including the One-Target condition), we found a main effect of condition (*F*_(3,5724)_ = 8.89, *P* < 0.00001), *f* = 0.07, with a *post hoc* test indicating a significant difference only between the three cognitive condition and the One-Target condition (Figure 4C). This result indicates that there is a clear effect of competition on the SP duration, but that this effect is independent of the nature of the distractor.

We then used a two-way ANOVA with the factors *Condition* and *Angle* and failed to find a main effect for any of those two factors (respectively, *F*_(2,4711)_ = 0.63, *P* = 0.56, *f* = 0.016 and *F*_(3,4711)_ = 0.56, *P* = 0.63), *f* = 0.02; Mean SP were almost identical (∼118 ms) for every cognitive and angular condition (Figures 4C,D). Based on the idea that RT is a good indicator of the level of competition involved, we divided the SP distribution of each condition according to RT into 7 non-overlapping time bins of 50 ms sorted by ascending order (Figure 4E). This Bin size was obtained using the automatic binning algorithm provided by Matlab and the associated function “histogram” which is based on the Scott’s rule (Scott, 1979). A two-way ANOVA with task conditions (control, congruent, or incongruent) and RT bins as factors was performed. The shorter and longer RT trials were excluded from this analysis (and not displayed on Figure 4E and on Supplementary Figure 3C) because of too small number to allow a comparison between the three conditions. This analysis revealed a main effect for bins (*F*_(6,4376)_ = 25.84, *P* < 0.00001), *f* = 0.19, but not for condition factor (*F*_(2,4376)_ = 2.27, *P* = 0.1), *f* = 0.03, indicating a gradual increase of the SP from the fastest to the slowest RT, consistent with an increased gabaergic activity of the cortico-spinal neurons activated during the voluntary movement. We then performed a one-way ANOVA with the factor condition (control, congruent, and incongruent) for each individual RT bin, and found a clear influence of cognitive condition for the two bins centered approximately on the mean RT of incongruent trials (*F*_(2,1113)_ = 6.03, *P* < 0.001), *f* = 0.13. This was confirmed by *post hoc* comparisons, which showed that SPs were significantly shorter in the incongruent condition (115.9 ± 1.8 ms) than in the congruent (124.6 ± 2 ms) and control conditions (125.3 ± 1.9 ms), indicating a reduction (or an inhibition) of cortical inhibition specifically for incongruent trials (Figure 4E, right inset). Importantly, a significant reduction of inhibition was also found for the SP recorded after stimulation with the figure-of-eight coil (i.e., for the earliest part of the SP) at roughly the same RT bins, attesting of the specificity of this cortical reduction in inhibition for incongruent trials with long RT (Supplementary Figure 3C). Critically, however, for this figure-of-eight set of data with shorter overall SPs, we did not find the linear increase from the faster to the slower RT bins (Supplementary Figure 3C), a result which could be interpreted in favor of a mainly spinal origin of this early SP. Finally, it is likely that in both experiments, the longer RT bins corresponded to outliers trials, with a mixed influence of task difficulty and decrease level of general attention over the course of almost 1,000 trials performed. We were therefore reluctant to interpret SP for those trials that were probably impacted by a lack of attention.

## Discussion

In order to better understand the neuronal bases of voluntary movement and its control, we designed a directional analog of the Stroop task (Stroop, 1935; MacLeod, 1991) allowing (1) to involve complex movements requiring several muscles, (2) to control the level of competition between simultaneous movement options, and (3) to clarify the functional role of inhibitory circuits in competition resolution, more particularly during the ongoing evolution of the decision.

Our DSLT task allowed us to generate the well-known three conditions of a classical Stroop task (control, congruent and incongruent), but also, to intermingle different motor context by varying both the orientation of the target and the relative position between the target and the distractor (Figure 1).

As expected, we found the classical results described in every conflict task, with longer RT for incongruent condition and a shorter RT for congruent condition relative to control condition (Figure 2A), which confirms the existence of both *behavioral congruency and interference effects* (MacLeod, 1991). As previously observed, the color dimension provided salient but irrelevant information which strongly influences the decision (Michelet et al., 2016). Moreover, the angular distance was also found to influence significantly the reaction times, with RT increasing with angular distance from 45 to 180° (Figure 2B), in line with the trivial idea that it is more difficult to process simultaneously information that are not close to each other. However, this is probably not a consequence of a facility to move the eyes from one potential target to the other as proposed by a study indicating that a closer distance do not imply purely attentional effects (Bock and Eversheim, 2000). Rather, we found that these results fit well with a model of interaction within a population of motor neurons, which predicts that the strength of the competition between two targets should be greater when they are far apart from each other than when they are close together (Cisek, 2006; Cisek, 2012), in agreement with *the affordance competition hypothesis* (Cisek and Pastor-Bernier, 2014). The combination of cognitive conditions and angular distances provided 12 different types of trials that could be ordered according to a continuum of increasing RT, which would be proportional to the level of competition or task difficulty (Figure 2C). Because the time of movement onset is given by the RT, this continuum of increasing RT clearly suggests that the decision to move is a function of a response activation (or decision) variable that depends on the gradual accumulation of evidence over time as predicted by several accumulator models (Gold and Shadlen, 2007; Thura, 2016). Hence it is likely that most of the competition resolution operates before movement execution, a process that engage several brain structures from prefrontal areas (Michelet et al., 2016) to the primary motor cortex (Michelet et al., 2010). However, it has been proposed that decision could still evolve after action onset (Tipper et al., 2000; Cisek, 2006; Resulaj et al., 2009; Cisek, 2012; Lepora and Pezzulo, 2015; Michalski et al., 2020). The analyses of the initial deviation (ID) indicate clearly that the decision process is not ended at the time of movement onset, and that decision variables still inform the executive brain regions during movement execution. Indeed, the deviations of reaching trajectories were significantly impacted by the cognitive conditions (Figure 3), and we found for the ID the same *congruency and interference effects* as described for RT. Regarding the angular distance analysis, the initial deviation is more pronounced for an angular separation of 90° between target and distractor, consistent with the vector geometry of reaching in a 2D plane. For the same reason, it was also expected that the initial deviation is less important in the 180° angle separation. At first sight, this later result could seem at odd with the stronger impact found on RT for angular separation of 180°. Our interpretation is that this is in accordance with previous works clearly showing that decisions take also biomechanical costs into account when choosing between multiple actions, and that these biomechanical costs bias movement choice before movement onset (Cos et al., 2011). Relative to the one-Target condition, we can then conclude that the movement trajectory is deviated toward the distractor in every condition (control, congruent, or incongruent), indicating that the representation of the task-irrelevant information (the distractor) is not totally suppressed at the time of movement onset. This is in agreement with the idea that several plans of action are prepared before actually choosing one of them, and that these potential actions are still present and competing after movement onset. In other words, when a competition exists between multiple potential targets, the unselected action program is not completely suppressed at movement onset (Cisek, 2012).

Because in every cognitive condition a clear influence of the competing response for the distractor is still present at least 100 ms after the start of the movement, a continuing process is probably still needed to finally reach the correct target. This process could involve the inhibition of the distractor, the enhancement of the activity related to the chosen target, or both. Although conflict resolution has been proposed to occur through cortical amplification of task-relevant information (Egner and Hirsch, 2005), it remains that “Response inhibition is the most basic form of behavioral control” (Stuphorn, 2015), and should still be considered. The continuum found in RT and ID for our 12 conditions (Figures 2C, 3C) confirms that this task is well suited to assess the involvement of inhibitory process in response competition because it allows to compare SP durations among a wide range of competition level.

Based on the report that RT and ID reflect, respectively, the pre- and post-movement competition between options, we tried to corelate these results with measures of SP that is known to provide a quantitative assessment of inhibitory processes. This SP could be separated into an early component thought to reflect spinal inhibition, and a late component determining the duration of the whole SP, involving inhibitory effects at cortical level (Ziemann et al., 1993; Chen et al., 1999). However, it has also been proposed that the SP is exclusively of cortical origin (Schnitzler and Benecke, 1994). In any case, it seems that SP is proportional to the amount of inhibitory inputs onto the M1 projection neurons (Taylor et al., 1997). Because the reaching movements are subserved by the activity of a population of neurons in the primary motor cortex, each of them being involved in several movement directions (Georgopoulos et al., 1986), we computed the SP for the 8 recorded muscles in order to reflect global (population level) inhibition during arm reaching movements (see Supplementary Figure 2). This is further recommended (1) because inhibitory influences responsible for the SP are widely distributed across muscles, irrespectively of their agonist or antagonistic role (Ho et al., 1998); (2) because agonist and antagonist could nevertheless be simultaneously activated (Latash, 2018).

Using the focal figure of 8 coil, we found relatively short SP (around 60 ms; see Supplementary Figure 2), indicating either an insufficient intensity of TMS pulse, or a spinal contribution to the recorded SP (Säisänen et al., 2008). With such coil, the area of stimulation is probably too focal and covers small surface of the M1 upper-limb area and may thus mostly reflect spinal mechanisms (Säisänen et al., 2008). Using the circular coil, allowing to use stronger stimulus intensity targeting a broader cortical region we recorded SP duration longer than 100 ms, and therefore more likely to depend on cortical GABAergic inhibition (Chen et al., 1999; Orth and Rothwell, 2004). This result is also in accordance with the well documented effect of simulation intensity on the SP duration (Taylor et al., 1997; Säisänen et al., 2008).

We first studied the SP as a function of cognitive conditions and angular distances. We found a significant difference between the One-Target and the cognitive conditions, indicating that the very presence of a competition modulate the SP duration. However, we failed to find an overall difference between the three cognitive conditions (Figure 4C).

We then speculate that if the SP is directly related to the competition, it should be positively correlated with the RT, irrespective of the cognitive condition. We then compare the SP for seven successive RT bins, thereby matching the RT for each condition (Figure 4E). The interaction between condition and RT bins is not significant, as well as the main effect of condition. However, we found a significant main effect of RT bins, with a positive correlation between RT and SP duration, suggesting that situations involving more competition also involves more inhibition. This is consistent with several experimental and theoretical observations, described below, which all lead to sustain our proposal.

This is firstly consistent with the accumulator models postulating that selection between multiple options operates through biased competition involving a mutual inhibition process between different brain regions or within M1. Indeed, we have previously shown that in parallel with the growing agonist activity for the target related movement, the activity related to the alternative movement toward the distractor is gradually inhibited during the RT period (Michelet et al., 2010). The increased inhibition found in the present experiment is consistent with this previous finding, and indicate with a direct measure of the inhibitory process that the inhibition could last after movement onset. Critically, a significant proportion of corticospinal neurons become active after EMG onset (Cheney and Fetz, 1980) and could directly benefit from this late process. Secondly, this is consistent with the proposal that selective inhibition build up progressively (Ridderinkhof et al., 2004), which implies that the strength of the inhibition should be greater for longer RT. This is even more likely because the SP was measured here just few milliseconds after movement onset, and consequently before the whole system returned to a baseline level of activation. In this respect, the SP is considered as a continuation of the mechanisms that led to initial choice (van den Berg et al., 2016).

Third, the build-up of the SP is consistent with a pharmacological experiment effect showing a GABAb agonist (Baclofen) dose-dependent increased duration of the SP (Siebner et al., 1998), thereby confirming the hypothesis of a gradual increase of the inhibitory processes.

However, at the neuronal level, a specificity emerges from the conflict situation beyond the strict competition: while a global increase of the inhibitory processes is found, correlatively with the increasing difficulty of the task, a drop in the inhibitory processes become visible for (late) incongruent trials (Figure 4E). This is consistent with previous report indicating that in difficult tasks, online error correction latencies (i.e., correction during ongoing movements) increases as the RT increases (Rabbitt and Vyas). This result is also in accordance with the activation-suppression hypothesis (Ridderinkhof et al., 2004), which predicts that long delay could imply a strong inhibition of both congruent and incongruent stimuli, which could impede the ability to finally choose any option. This hypothesis is strengthened by the broad tuning in population coding of the primary motor cortex, implying that the same neurons could be involved in different reaching movements. Indeed, the inhibition of the movement toward the distractor could hence possibly affect neurons involved in both reaching actions (i.e., toward target and distractor). In this context, a dampening of the inhibition could momentarily impede the process of action selection, and allow other processes to bias decision through vacillation between the two options, also providing more time to complete the decision process. This result is in accordance with the seemingly paradoxical increase of motor variability that were previously found to improve learning performance (Wu et al., 2014). Furthermore, and considering the gabaergic origin of the SP, it is noteworthy that an administration of baclofen (GABAb agonist) also increases behavioral flexibility (Beas et al., 2016).

We hence propose that this transient reduction of GABAergic inhibition could serve as an adaptive process to let the system free of changing its mind until the last moment before reaching the target. This result is consistent with previous work demonstrating that under conflicting or uncertain conditions, the motor system adapts quickly to a changing and unpredictable context by equalizing the preparation of alternative responses (Bosc et al., 2021). In accordance with this hypothesis, the reach kinematics indicate a larger proportion of trajectories with a change of direction for these trials, confirming a greater disposition to vacillation or changing mind strategy (Supplementary Figure 4).

We could link this effect to an uncertainty that increases as time elapses, and a more likely influence of upstream brain structures to influence this process. This is partly confirmed by the fact that in incongruent trials, the reaching movement is first directed toward a position intermediate between the target and the distractor. Vacillation here resemble the exploration mode favored by an increased baseline release of noradrenaline making neurons more responsive to any stimulus, thereby allowing a broad scan of possible options (Aston-Jones and Cohen, 2005). Interestingly, anterior midcingulate (aMCC) inputs are supposed to drive these exploration mode (Aston-Jones and Cohen, 2005), and this brain regions send also direct inputs to both the spinal cord and M1, which could explain, respectively the decrease of the early (Supplementary Figure 3) and late (Figure 4) SP. Previous results in monkeys, in a conflict task where vacillation or self-corrected movements were found, indicated that the timing of aMCC activation for incongruent trails is compatible with the timing of the SP reduction (Michelet et al., 2016).

The present experiment used a wide range of movement directions and angles between target and distractor that have somehow limited the number of trials for each condition and consequently the power of our statistical analysis. Moreover, we have explored the validity of two types of coils, which have also limited the number of subjects we were able to consider simultaneously. In future experiments following this pilot study, we will focus our analysis on fewer movement directions, using exclusively the circular coil. Such evolution of our experimental protocol would allow to directly test, with more participants, the interaction between M1 and other frontal areas (PMd, SMA) using the dual-coil TMS technic during movement execution.

Indeed, other regions of the frontal cortex are also likely to participate in the inhibition of M1, such as pre-SMA (Duque et al., 2013; Quoilin et al., 2021), or the Pre-motor cortex (Parmigiani et al., 2015; Parmigiani et al., 2018) in line with the idea that inhibition is a fundamental function of the frontal cortex (but see Neige et al., 2021 for a comprehensive review). This idea is also well in line with the fact that there are several possibilities of inhibition within M1 (Sanger et al., 2001), potentially implemented by different brain regions, either cortical or subcortical. For instance, one such candidate is the subthalamic nucleus, a basal ganglia region well known to be involved in the inhibition of competing motor representation, acting as a brake on the cortico-striatal system (Cavanagh et al., 2011), thereby preventing premature responding (Frank, 2006; Cavanagh et al., 2011; Mirabella et al., 2012; Zavala et al., 2015; Wessel et al., 2019) to facilitate decision making under conflict (Cavanagh et al., 2011; Mirabella et al., 2012; Zavala et al., 2015).

Overall, this pilot study provides evidence that a subtle imbalance of the GABAergic inhibitory processes participates, even after movement onset, to the conflict-resolution. This process could complete the repertoire of adaptive strategies allowing before (Frank, 2006; Cavanagh et al., 2011; Mirabella et al., 2012; Duque et al., 2013; Klein et al., 2014), during and after (Bosc et al., 2021) movement control over competition and more specifically conflict situations.

## Data Availability Statement

The raw data supporting the conclusions of this article will be made available by the authors, without undue reservation.

## Ethics Statement

The studies involving human participants were reviewed and approved by the CPP N° 2013A01444936. The patients/participants provided their written informed consent to participate in this study.

## Author Contributions

TM conceived and designed the experiments. NL and MG programmed the software. BR and AD collected the data. BR, AR, and TM conducted the data analyses and wrote the manuscript. All authors contributed to the article and approved the submitted version.

## Conflict of Interest

The authors declare that the research was conducted in the absence of any commercial or financial relationships that could be construed as a potential conflict of interest.

## Publisher’s Note

All claims expressed in this article are solely those of the authors and do not necessarily represent those of their affiliated organizations, or those of the publisher, the editors and the reviewers. Any product that may be evaluated in this article, or claim that may be made by its manufacturer, is not guaranteed or endorsed by the publisher.

## References

[B1] Aston-Jones G. Cohen J. D. (2005). An integrative theory of locus coeruleus-norepinephrine function: adaptive gain and optimal performance. *Annu. Rev. Neurosci.* 28 403–450. 16022602 10.1146/annurev.neuro.28.061604.135709

[B2] Bari A. Robbins T. W. (2013). Inhibition and impulsivity: behavioral and neural basis of response control. *Progr. Neurobiol.* 108 44–79. 10.1016/j.pneurobio.2013.06.005 23856628

[B3] Beas B. S. Setlow B. Bizon J. L. (2016). Effects of acute administration of the GABA(B) receptor agonist baclofen on behavioral flexibility in rats. *Psychopharmacology (Berl.)* 233 2787–2797. 10.1007/s00213-016-4321-y 27256354 PMC4919234

[B4] Bock O. Eversheim U. (2000). The mechanisms of movement preparation: a precuing study. *Behav. Brain Res.* 108 85–90. 10.1016/s0166-4328(99)00134-5 10680760

[B5] Bosc M. Bucchioni G. Ribot B. Michelet T. (2021). Bypassing use-dependent plasticity in the primary motor cortex to preserve adaptive behavior. *Sci. Rep.* 11:12102. 10.1038/s41598-021-91663-9 34103649 PMC8187343

[B6] Botvinick M. M. Cohen J. D. Carter C. S. (2004). Conflict monitoring and anterior cingulate cortex: an update. *Trends Cogn. Sci.* 8 539–546. 10.1016/j.tics.2004.10.003 15556023

[B7] Cavanagh J. F. Wiecki T. V. Cohen M. X. Figueroa C. M. Samanta J. Sherman S. J. (2011). Subthalamic nucleus stimulation reverses mediofrontal influence over decision threshold. *Nat. Neurosci.* 14 1462–1467. 10.1038/nn.2925 21946325 PMC3394226

[B8] Chen R. Lozano A. M. Ashby P. (1999). Mechanism of the silent period following transcranial magnetic stimulation. Evidence from epidural recordings. *Exp. Brain Res. Experimentelle Hirnforschung Expérimentation cérébrale* 128 539–542. 10.1007/s002210050878 10541749

[B9] Cheney P. D. Fetz E. E. (1980). Functional classes of primate corticomotoneuronal cells and their relation to active force. *J. Neurophysiol.* 44 773–791. 10.1152/jn.1980.44.4.773 6253605

[B10] Cisek P. (2006). Integrated neural processes for defining potential actions and deciding between them: a computational model. *J. Neurosci.* 26 9761–9770. 10.1523/JNEUROSCI.5605-05.2006 16988047 PMC6674435

[B11] Cisek P. (2012). Making decisions through a distributed consensus. *Curr. Opin. Neurobiol.* 22 927–936. 10.1016/j.conb.2012.05.007 22683275

[B12] Cisek P. Pastor-Bernier A. (2014). On the challenges and mechanisms of embodied decisions. *Philos. Trans. R. Soc. Lond. Ser. B Biol. Sci.* 369:20130479. 10.1098/rstb.2013.0479 25267821 PMC4186232

[B13] Coallier E. Michelet T. Kalaska J. F. (2015). Dorsal premotor cortex: neural correlates of reach target decisions based on a color-location matching rule and conflicting sensory evidence. *J. Neurophysiol.* 113 3543–3573. 10.1152/jn.00166.2014 25787952 PMC4461887

[B14] Cohen J. (1988). *Statistical Power Analysis for the Behavioral Sciences.* Hillsdale, NJ: L. Erlbaum Associates.

[B15] Cos I. Bélanger N. Cisek P. (2011). The influence of predicted arm biomechanics on decision making. *J. Neurophysiol.* 105 3022–3033. 10.1152/jn.00975.2010 21451055

[B16] Cos I. Medleg F. Cisek P. (2012). The modulatory influence of endpoint controllability on decisions between actions. *J. Neurophysiol*. 108 1764–1780. 10.1152/jn.00081.2012 22773776

[B17] Duque J. Olivier E. Rushworth M. (2013). Top-Down inhibitory control exerted by the medial frontal cortex during action selection under conflict. *J. Cogn. Neurosci.* 25 1634–1648. 10.1162/jocn_a_00421 23662862

[B18] Egner T. Hirsch J. (2005). Cognitive control mechanisms resolve conflict through cortical amplification of task-relevant information. *Nat. Neurosci.* 8 1784–1790. 10.1038/nn1594 16286928

[B19] Frank M. J. (2006). Hold your horses: a dynamic computational role for the subthalamic nucleus in decision making. *Neural Netw.* 19 1120–1136. 10.1016/j.neunet.2006.03.006 16945502

[B20] Georgopoulos A. P. (2000). Neural aspects of cognitive motor control. *Curr. Opin. Neurobiol.* 10 238–241. 10.1016/s0959-4388(00)00072-6 10753794

[B21] Georgopoulos A. P. Schwartz A. B. Kettner R. E. (1986). Neuronal population coding of movement direction. *Science* 233 1416–1419. 3749885 10.1126/science.3749885

[B22] Gold J. Shadlen M. (2007). The neural basis of decision making. *Annu. Rev. Neurosci.* 30 535–574. 17600525 10.1146/annurev.neuro.29.051605.113038

[B23] Ho K. H. Nithi K. Mills K. R. (1998). Covariation between human intrinsic hand muscles of the silent periods and compound muscle action potentials evoked by magnetic brain stimulation: evidence for common inhibitory connections. *Exp. Brain Res. Experimentelle Hirnforschung Expérimentation cérébrale* 122 433–440. 10.1007/s002210050531 9827862

[B24] Klein P.-A. Olivier E. Duque J. (2012). Influence of reward on corticospinal excitability during movement preparation. *J. Neurosci.* 32 18124–18136. 10.1523/JNEUROSCI.1701-12.2012 23238727 PMC6621748

[B25] Klein P.-A. Petitjean C. Olivier E. Duque J. (2014). Top-down suppression of incompatible motor activations during response selection under conflict. *Neuroimage* 86 138–149. 10.1016/j.neuroimage.2013.08.005 23939021

[B26] Latash M. L. (2018). Muscle coactivation: definitions, mechanisms, and functions. *J. Neurophysiol.* 120 88–104. 10.1152/jn.00084.2018 29589812 PMC6093955

[B27] Leocani L. Cohen L. G. Wassermann E. M. Ikoma K. Hallett M. (2000). Human corticospinal excitability evaluated with transcranial magnetic stimulation during different reaction time paradigms. *Brain* 123(Pt 6) 1161–1173. 10.1093/brain/123.6.1161 10825355

[B28] Lepora N. F. Pezzulo G. (2015). Embodied choice: how action influences perceptual decision making. *PLoS Comp. Biol.* 11:e1004110. 10.1371/journal.pcbi.1004110 25849349 PMC4388485

[B29] Ludwig C. J. H. Gilchrist I. D. (2002). Measuring saccade curvature: a curve-fitting approach. *Behav. Res. Methods Instrum. Comput.* 34 618–624. 10.3758/bf03195490 12564565

[B30] MacLeod C. M. (1991). Half a century of research on the Stroop effect: an integrative review. *Psychol. Bull.* 109 163–203. 10.1037/0033-2909.109.2.163 2034749

[B31] Meyer D. E. Osman A. M. Irwin D. E. Yantis S. (1988). Modern mental chronometry. *Biol. Psychol.* 26 3–67. 10.1016/0301-0511(88)90013-0 3061480

[B32] Michalski J. Green A. M. Cisek P. (2020). Reaching decisions during ongoing movements. *J. Neurophysiol.* 123 1090–1102. 10.1152/jn.00613.2019 32049585 PMC7099481

[B33] Michelet T. Bioulac B. Langbour N. Goillandeau M. Guehl D. Burbaud P. (2016). Electrophysiological correlates of a versatile executive control system in the monkey anterior cingulate cortex. *Cereb. Cortex* 26 1684–1697. 10.1093/cercor/bhv004 25631057

[B34] Michelet T. Duncan G. H. Cisek P. (2010). Response competition in the primary motor cortex: corticospinal excitability reflects response replacement during simple decisions. *J. Neurophysiol.* 104 119–127. 10.1152/jn.00819.2009 20445034

[B35] Mirabella G. Iaconelli S. Romanelli P. Modugno N. Lena F. Manfredi M. (2012). Deep brain stimulation of subthalamic nuclei affects arm response inhibition in Parkinson&apos;s patients. *Cereb. Cortex* 22 1124–1132. 10.1093/cercor/bhr187 21810782

[B36] Neige C. Rannaud Monany D. Lebon F. (2021). Exploring cortico-cortical interactions during action preparation by means of dual-coil transcranial magnetic stimulation: a systematic review. *Neurosci. Biobehav. Rev.* 128 678–692. 10.1016/j.neubiorev.2021.07.018 34274404

[B37] Oldfield R. C. (1971). The assessment and analysis of handedness: the Edinburgh inventory. *Neuropsychologia* 9 97–113. 10.1016/0028-3932(71)90067-4 5146491

[B38] Orth M. Rothwell J. C. (2004). The cortical silent period: intrinsic variability and relation to the waveform of the transcranial magnetic stimulation pulse. *Clin. Neurophysiol. : Off. J. Int. Federation Clin. Neurophysiol.* 115 1076–1082. 10.1016/j.clinph.2003.12.025 15066533

[B39] Parmigiani S. Barchiesi G. Cattaneo L. (2015). The dorsal premotor cortex exerts a powerful and specific inhibitory effect on the ipsilateral corticofacial system: a dual-coil transcranial magnetic stimulation study. *Exp. Brain Res.* 233 3253–3260. 10.1007/s00221-015-4393-7 26233241

[B40] Parmigiani S. Zattera B. Barchiesi G. Cattaneo L. (2018). Spatial and temporal characteristics of set-related inhibitory and excitatory inputs from the dorsal premotor cortex to the ipsilateral motor cortex assessed by dual-coil transcranial magnetic stimulation. *Brain Topogr.* 31 795–810. 10.1007/s10548-018-0635-x 29460169

[B41] Quoilin C. Dricot L. Genon S. De Timary P. Duque J. (2021). Neural bases of inhibitory control: combining transcranial magnetic stimulation and magnetic resonance imaging in alcohol-use disorder patients. *Neuroimage* 224:117435. 10.1016/j.neuroimage.2020.117435 33039622

[B42] Rabbitt P. Vyas S. (1981). Processing a display even after you make a response to it. How perceptual errors can be corrected. *Q. J. Exp. Psychol. Sect. A* 33 223–239. 10.1080/14640748108400790

[B43] Resulaj A. Kiani R. Wolpert D. M. Shadlen M. N. (2009). Changes of mind in decision-making. *Nature* 461 263–266. 19693010 10.1038/nature08275 PMC2875179

[B44] Ridderinkhof K. R. Van Den Wildenberg W. P. Wijnen J. G. Burle B. (2004). “Response inhibition in conflict tasks is revealed in delta plots,” in *Cognitive Neuroscience of Attention*, ed. Posner M. I. (New York, NY: Guilford Press), 369–377.

[B45] Rossi S. Hallett M. Rossini P. M. Pascual-Leone A. Safety of Tms Consensus Group. (2009). Safety, ethical considerations, and application guidelines for the use of transcranial magnetic stimulation in clinical practice and research. *Clin. Neurophysiol*. 120 2008–2039. 19833552 10.1016/j.clinph.2009.08.016 PMC3260536

[B46] Säisänen L. Pirinen E. Teitti S. Könönen M. Julkunen P. Määttä S. (2008). Factors influencing cortical silent period: optimized stimulus location, intensity and muscle contraction. *J. Neurosci. Methods* 169 231–238. 10.1016/j.jneumeth.2007.12.005 18243329

[B47] Sanger T. D. Garg R. R. Chen R. (2001). Interactions between two different inhibitory systems in the human motor cortex. *J. Physiol. (Lond.)* 530 307–317. 10.1111/j.1469-7793.2001.0307l.x 11208978 PMC2278414

[B48] Schnitzler A. Benecke R. (1994). The silent period after transcranial magnetic stimulation is of exclusive cortical origin: evidence from isolated cortical ischemic lesions in man. *Neurosci. Lett.* 180 41–45. 10.1016/0304-3940(94)90909-1 7877758

[B49] Scott D. W. (1979). On optimal and data-based histograms. *Biometrika* 66 605–610.

[B50] Siebner H. R. Dressnandt J. Auer C. Conrad B. (1998). Continuous intrathecal baclofen infusions induced a marked increase of the transcranially evoked silent period in a patient with generalized dystonia. *Muscle Nerve* 21 1209–1212. 10.1002/(sici)1097-4598(199809)21:9<1209::aid-mus15>3.0.co;2-m 9703450

[B51] Smith E. E. (1968). Choice reaction time: an analysis of the major theoretical positions. *Psychol. Bull.* 69 77–110. 10.1037/h0020189 4867597

[B52] Stroop J. R. (1935). Studies of interference in serial verbal reactions. *J. Exp. Psychol.* 18 643–662.

[B53] Stuphorn V. (2015). Neural mechanisms of response inhibition. *Curr. Opin. Behav. Sci.* 1, 64–71.

[B54] Taylor J. L. Allen G. M. Butler J. E. Gandevia S. C. (1997). Effect of contraction strength on responses in biceps brachii and adductor pollicis to transcranial magnetic stimulation. *Exp. Brain Res.* 117 472–478. 10.1007/s002210050243 9438716

[B55] Thura D. (2016). How to discriminate conclusively among different models of decision making? *J. Neurophysiol.* 115 2251–2254. 10.1152/jn.00911.2015 26538611 PMC4922450

[B56] Tipper S. P. Howard L. A. Houghton G. (2000). “Behavioural consequences of selection from neural population codes,” in *Control of Cognitive Processes: Attention and Performance XVIII*, eds Monsell S. Driver J. (Cambridge, MA: MIT Press), 223–245.

[B57] van den Berg R. Anandalingam K. Zylberberg A. Kiani R. Shadlen M. N. Wolpert D. M. (2016). A common mechanism underlies changes of mind about decisions and confidence. *Elife* 5:e12192. 10.7554/eLife.12192 26829590 PMC4798971

[B58] Wessel J. R. Waller D. A. Greenlee J. D. (2019). Non-selective inhibition of inappropriate motor-tendencies during response-conflict by a fronto-subthalamic mechanism. *Elife* 8:e42959. 10.7554/eLife.42959 31063130 PMC6533064

[B59] World Medical Association General Assembly [WMAGA] (2008). Declaration of Helsinki. Ethical principles for medical research involving human subjects (6th revision). *World Med. J.* 54 122–125. 19886379

[B60] Wu H. G. Miyamoto Y. R. Castro L. N. G. Olveczky B. P. Smith M. A. (2014). Temporal structure of motor variability is dynamically regulated and predicts motor learning ability. *Nat. Neurosci*. 17 312–321. 10.1038/nn.3616 24413700 PMC4442489

[B61] Zavala B. Zaghloul K. Brown P. (2015). The subthalamic nucleus, oscillations, and conflict. *Mov. Disord.* 30 328–338. 10.1002/mds.26072 25688872 PMC4357561

[B62] Ziemann U. Netz J. Szelényi A. Hömberg V. (1993). Spinal and supraspinal mechanisms contribute to the silent period in the contracting soleus muscle after transcranial magnetic stimulation of human motor cortex. *Neurosci. Lett.* 156 167–171. 10.1016/0304-3940(93)90464-v 8414181

