# Supplementary material for: Competition, Conflict and Change of Mind: A Role of GABAergic Inhibition in the Primary Motor Cortex
Source: Front Hum Neurosci. 2022 Jan 4;15:736732. doi: 10.3389/fnhum.2021.736732 (PMC8763692; doi:10.3389/fnhum.2021.736732)
Supplement: Supplementary file 1 [file Data_Sheet_1.PDF]

Competition, conflict and change of mind: a role of GABAergic inhibition in the primary motor cortex

Supplementary data:

- Supplemental figure 1
- Supplemental figure 2
- Supplemental figure 3
- Supplemental figure 4
- Supplemental figure 5
- Supplemental table 1

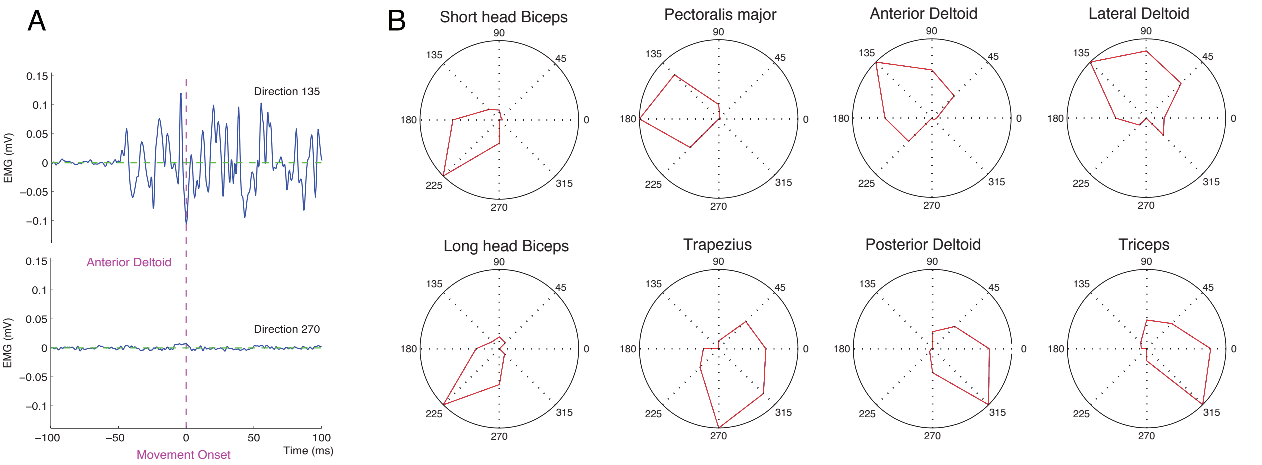

Supplemental figure 1: Muscle activation patterns

A) Surface electromyogram (EMG) activity of the anterior deltoid for reaching a target at 135° (top trace) or at 270° (bottom trace). Vertical dashed line indicates the movement onset. B) Spatial tuning of activity of 8 muscles of the right upper arm for reaching movements towards 8 different targets (one-target condition) arrayed on a circle at 45° increments. Polar plots give EMG activity during a 200 ms epoch centered on the movement onset, normalized with the 1s rest period EMG activity recorded at the very beginning of each trial.

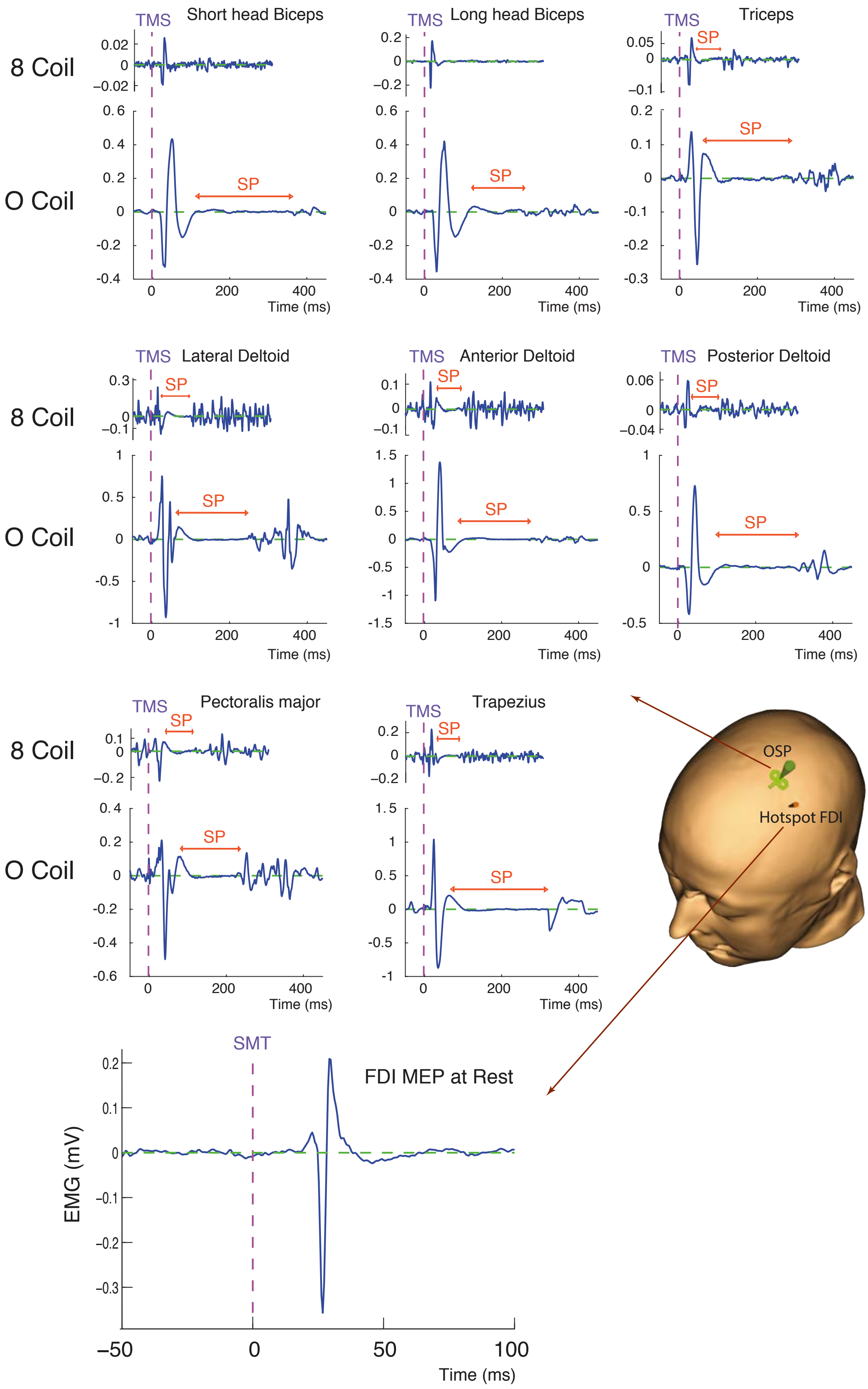

Supplemental figure 2: EMG responses to the figure-of-eight and circular coil TMS

A) SPs recorded in the same 8 muscles of the upper right arm in two representative subjects after either the 8 coil (upper trace) or O coil (lower trace) TMS. B) The stimulation areas corresponding to the FDI muscle hotspot and arm muscles optimal scalp position (OSP) are represented on a 3D-rendering of a subject as recorded by the navigated brain stimulation software (brainsight). C) Example of MEP measured from the FDI muscle of the right hand produced by the stimulation in order to estimate the resting motor threshold.

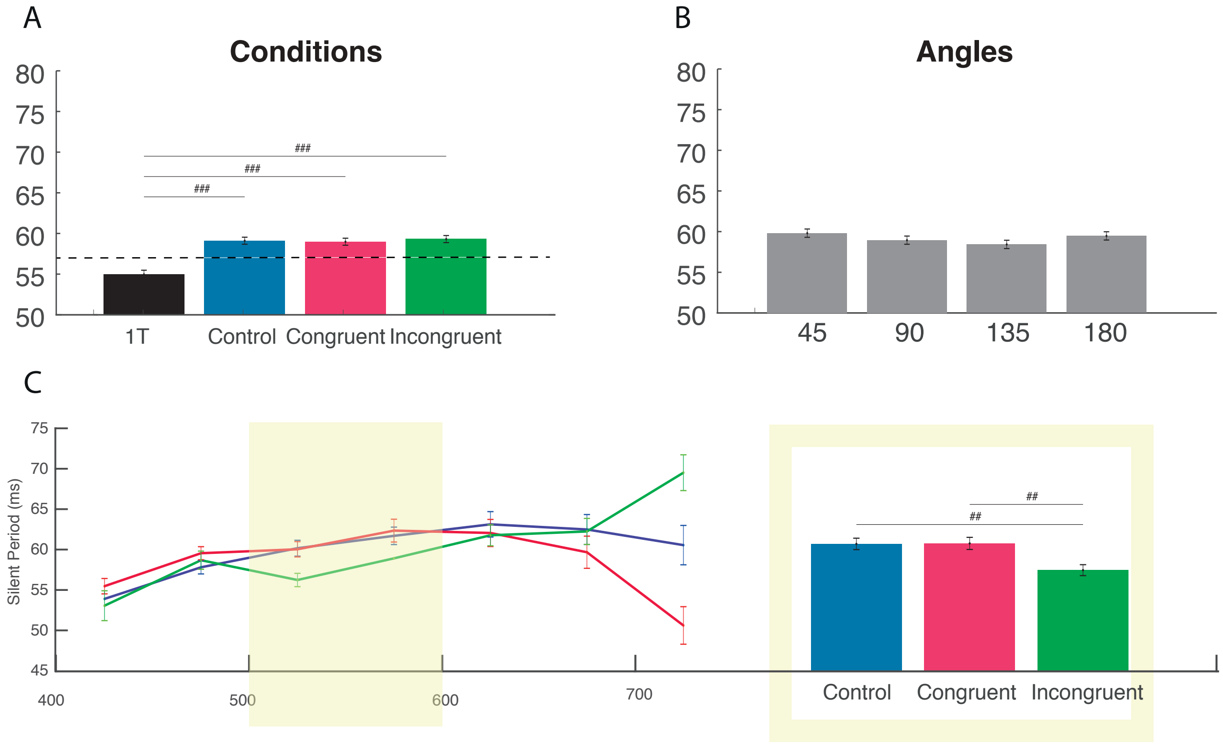

Supplemental figure 3: Silent Period Duration (SP) after circular coil TMS.

The SP duration is not modulated by the cognitive condition. B) The SP duration is not modulated by the angular distance between the target and distractor. C) Mean SP in each bin of RT as a function of cognitive condition (control, congruent and incongruent) showing an absence of modulation by the competition strength. SP analysis for the subgroup corresponding to the two bins highlighted in grey is presented in the adjacent inset (same ordinate axis as for the main graph).

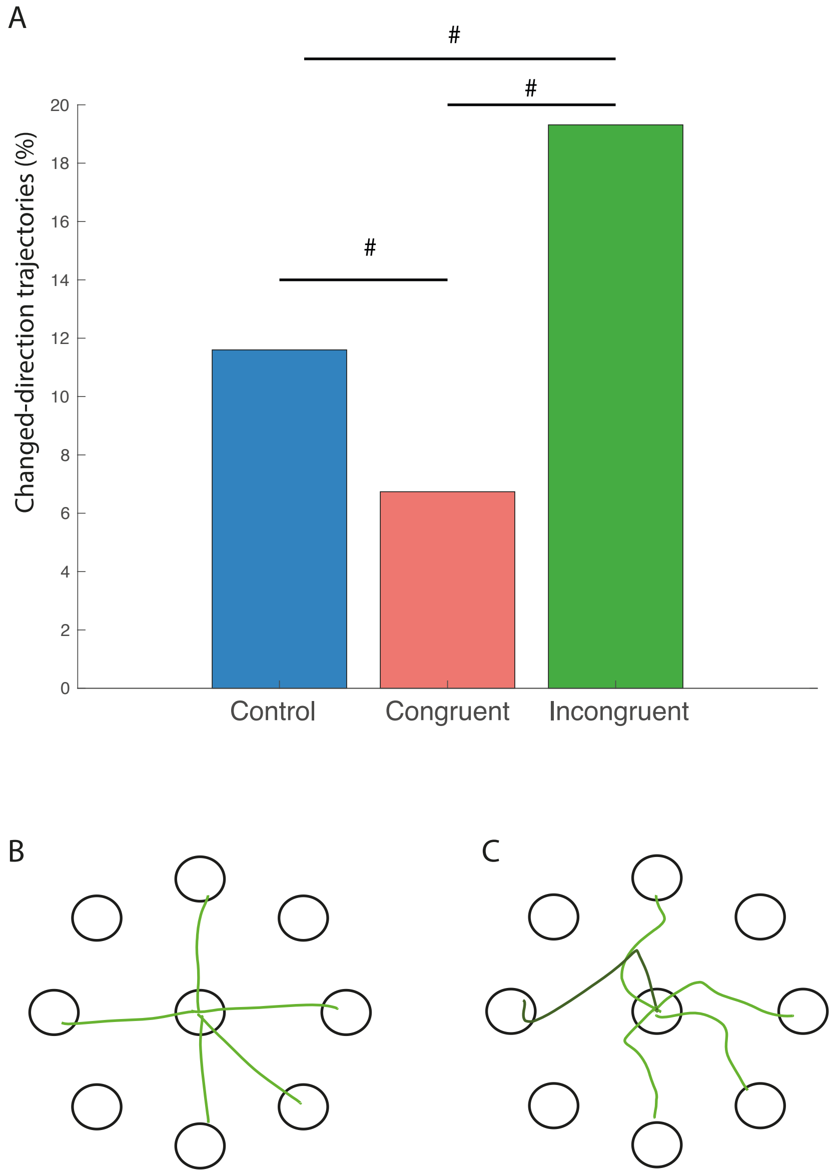

Supplemental figure 4: Example of change in reaching direction (vacillation)

A) Percentage of trials during which the hand trajectory changed course during the movement. While the majority of movements formed a single-curved trajectory from the central cue to the choice target, in a significantly greater proportion of incongruent trials (19.3 %, $\chi 2 = 14.6$, $P < 0.001$); congruent (6.7 %) ; control (11.6 %)) the hand trajectory changed course during the movement. B) Example of single-curved trajectories compared with C) double-curved reach trajectories in the same direction, indicating a change of mind between the onset and the end of the movement.

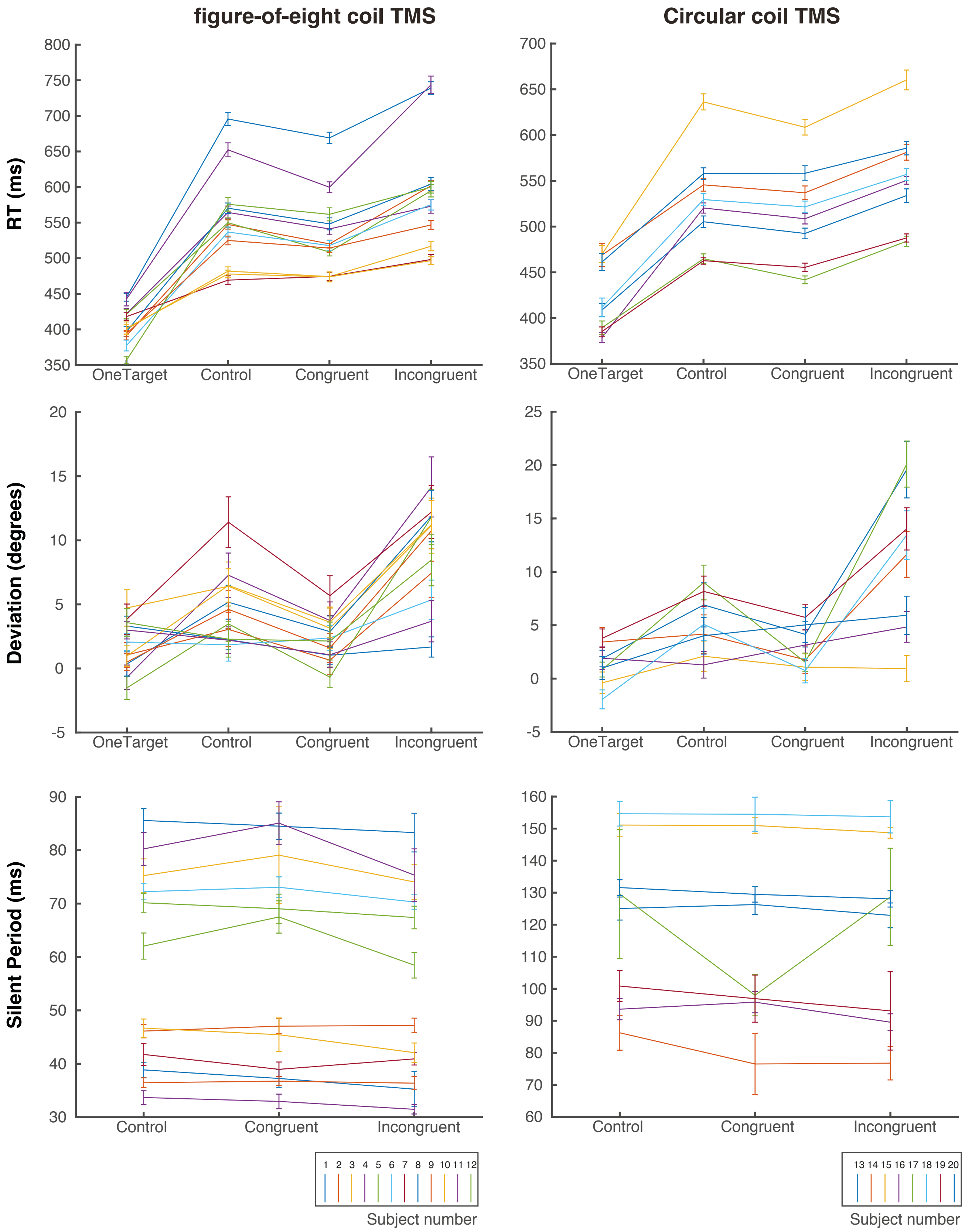

Supplemental figure 5: Individual data

Contribution of individual subjects for the Figure-of-eight coil experiment (left column) and the Circular coil experiment (right column). Subject numbers are coded with color.
First raw : Reaction time (RT); Second raw : Initial deviation (ID); Third raw : Silent Period Duration (SP).

	Direction (°)							
**Muscle**	**0**	**45**	**90**	**135**	**180**	**225**	**270**	**315**
**Lateral Deltoide**	80	85	95	90	90	60	80	85
**Short Head Biceps**	25	50	50	40	40	30	30	25
**Long Head Biceps**	45	45	55	60	55	60	45	50
**Triceps**	70	90	95	95	80	55	50	60
**Posterior Deltoide**	70	50	65	50	45	35	70	75
**Anterior Deltoide**	90	90	100	95	100	60	70	75
**Pectoralis Major**	45	55	70	90	95	85	40	35
**Trapezius**	100	55	35	35	65	60	100	100

Supplemental table 1: Muscle involved in SP calculation

Percentage of subjects for which a dedicated muscle was involved in the calculation of the global SP for any given movement direction.
